# STAGETOOL, a Novel Automated Approach for Mouse Testis Histological Analysis

**DOI:** 10.1210/endocr/bqac202

**Published:** 2022-12-03

**Authors:** Oliver Meikar, Daniel Majoral, Olli Heikkinen, Eero Valkama, Sini Leskinen, Ana Rebane, Pekka Ruusuvuori, Jorma Toppari, Juho-Antti Mäkelä, Noora Kotaja

**Affiliations:** Institute of Biomedicine and Translational Medicine, University of Tartu, 50411 Tartu, Estonia; Institute of Biomedicine, Integrative Physiology and Pharmacology Unit, University of Turku, 20520 Turku, Finland; Computational Neuroscience Lab, Institute of Computer Science, University of Tartu, 51014 Tartu, Estonia; Institute of Biomedicine, Integrative Physiology and Pharmacology Unit, University of Turku, 20520 Turku, Finland; Institute of Biomedicine, Integrative Physiology and Pharmacology Unit, University of Turku, 20520 Turku, Finland; Institute of Biomedicine, Integrative Physiology and Pharmacology Unit, University of Turku, 20520 Turku, Finland; Institute of Biomedicine and Translational Medicine, University of Tartu, 50411 Tartu, Estonia; Institute of Biomedicine, University of Turku, 20520 Turku, Finland; Institute of Biomedicine, Integrative Physiology and Pharmacology Unit, University of Turku, 20520 Turku, Finland; Department of Pediatrics, Turku University Hospital, 20520 Turku, Finland; Centre for Population Health Research, University of Turku and Turku University Hospital, 20520 Turku, Finland; Institute of Biomedicine, Integrative Physiology and Pharmacology Unit, University of Turku, 20520 Turku, Finland; Institute of Biomedicine, Integrative Physiology and Pharmacology Unit, University of Turku, 20520 Turku, Finland

**Keywords:** mouse testis histology, spermatogenesis, seminiferous epithelial cycle, automated analysis, deep learning, DAPI staining

## Abstract

Spermatogenesis is a complex differentiation process that takes place in the seminiferous tubules. A specific organization of spermatogenic cells within the seminiferous epithelium enables a synchronous progress of germ cells at certain steps of differentiation on the spermatogenic pathway. This can be observed in testis cross-sections where seminiferous tubules can be classified into distinct stages of constant cellular composition (12 stages in the mouse). For a detailed analysis of spermatogenesis, these stages have to be individually observed from testis cross-sections. However, the recognition of stages requires special training and expertise. Furthermore, the manual scoring is laborious considering the high number of tubule cross-sections that have to be analyzed. To facilitate the analysis of spermatogenesis, we have developed a convolutional deep neural network-based approach named “STAGETOOL.” STAGETOOL analyses histological images of 4′,6-diamidine-2′-phenylindole dihydrochloride (DAPI)-stained mouse testis cross-sections at ×400 magnification, and very accurately classifies tubule cross-sections into 5 stage classes and cells into 9 categories. STAGETOOL classification accuracy for stage classes of seminiferous tubules of a whole-testis cross-section is 99.1%. For cellular level analysis the F1 score for 9 seminiferous epithelial cell types ranges from 0.80 to 0.98. Furthermore, we show that STAGETOOL can be applied for the analysis of knockout mouse models with spermatogenic defects, as well as for automated profiling of protein expression patterns. STAGETOOL is the first fluorescent labeling–based automated method for mouse testis histological analysis that enables both stage and cell-type recognition. While STAGETOOL qualitatively parallels an experienced human histologist, it outperforms humans time-wise, therefore representing a major advancement in male reproductive biology research.

Sperm production in male mammals is highly efficient, with tens to hundreds of millions of spermatozoa produced every day. Spermatogenesis—male germ cell differentiation—occurs in the long convoluted seminiferous tubules inside the testis. The epithelium of seminiferous tubules contains several layers of differentiating germ cells and a lumen, where spermatozoa are released. Spermatogenesis is a complex mechanism, which starts with the propagation of spermatogonia via mitotic divisions, followed by meiosis of spermatocytes that generate haploid round spermatids (RS) with unique genomes, ending in the subsequent cellular differentiation into elongated spermatids. The spermatogenic cells spermatogonia, spermatocytes, and spermatids can be further divided into several subgroups based on their developmental phase. In contrast, Sertoli cells are the only somatic cell type within the seminiferous epithelium, and their primary function is to nurse and support the spermatogenic cells (1).

The fact that differentiation of 4 to 5 hierarchical generations of germ cells is synchronous and temporally controlled is a key to understanding testicular histology (2, 3). In many mammalian species, including the mouse, germ cells form concentric layers within a cross-section of a seminiferous tubule. The layer composition is predictable, and spermatids at a given developmental step are always found with particular types of spermatocytes and spermatogonia (Fig. 1). These cell associations are known as stages of the seminiferous epithelial cycle. In most mammalian species, including the mouse and human, 12 (I-XII) epithelial stages (Fig. 1) can be identified (4–7). The stages continuously cycle over time and form the wave of spermatogenesis along the seminiferous tubule. In the mouse, the stages are organized segmentally and can be identified easily, whereas in the human, the stages follow each other spirally, which complicates stage recognition in human testicular cross-sections.

**Figure 1.**
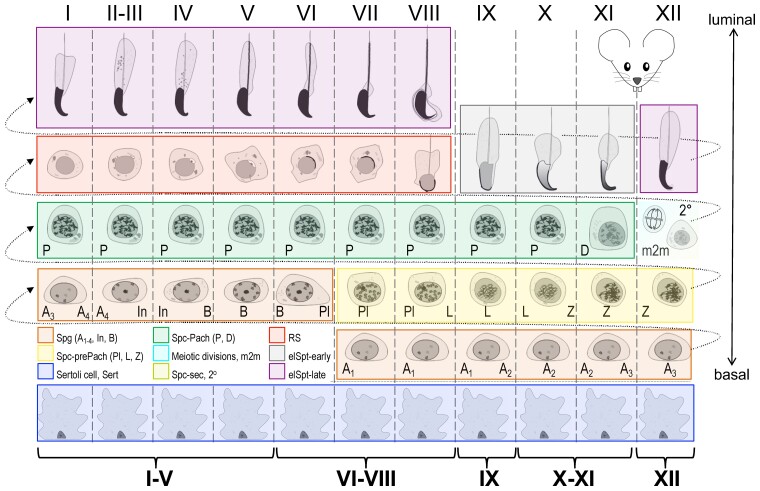
Spermatogenic cell differentiation and stages of the mouse seminiferous epithelial cycle. A spermatogonium that enters spermatogenesis has to pass through all the depicted developmental phases (from left to right, bottom to top) plus 6 mitotic and 2 meiotic divisions to be released as mature elongated spermatids. As spermatogenesis proceeds, spermatogenic cells move from the basal towards the luminal compartment. The progress of 4 to 5 generations of spermatogenic cells (in rows) is synchronized, and certain spermatids (highlighted with red, gray, and purple) are always associated with particular types of spermatocytes (green and yellow) and spermatogonia (orange); these are the 12 (I-XII; columns) stages of the seminiferous epithelial cycle. Spermatogonia (Spg; orange) exist as type A-undifferentiated plus A1 to A4, Intermediate (In) and type B. Primary spermatocytes can be divided into prepachytene (Spc-prePach; yellow, including preleptotene, Pl; leptotene, L; zygotene, Z), and pachytene (Spc-Pach; green) (pachytene, P; plus diplotene, D) spermatocytes. Meiotic divisions (m2m; cyan) and secondary spermatocytes (Spc-sec, 2^o^; olive) can be found solely in stage XII. Postmeiotic germ cell differentiation, that is, spermiogenesis, can be further divided into 16 steps (1-16); encompassing round spermatids (RS; red), and 2 categories of elongating spermatids: early (elSpt-early; gray) and late (elSpt-late; purple). Sertoli cells (Sert; blue).

Identification of the epithelial stage is essential for the analysis of spermatogenesis and male fertility. Most spermatogenesis-associated genes have a temporally restricted (*i.e.*, stage- and cell-type dependent) pattern of expression because the proteins they encode are needed at (a) specific step(s) of spermatogenic differentiation. Furthermore, Sertoli cells also function in a stage-dependent manner, because the needs of different germ cell combinations are distinct (3). Combining the knowledge of the spermatogenic cell type and the epithelial stage, where a protein is expressed, are the prerequisites to bring individual findings into a broader perspective. Moreover, only a stage-oriented approach can provide the means to comprehend the histopathology of infertile mouse models and aid in the analysis of the underlying causes and mechanisms. Given the constant decline of semen quality globally, there is an increasing demand for knowledge about the contributing factors (8) that basic research is able to reveal.

Stage recognition is primarily based on characteristics of postmeiotic elongating spermatids in any particular cross-section: namely their nuclear morphology, intraepithelial localization, and bundling (2, 6, 7). Changes in the morphology of spermatogenic cells across stages are gradual and subtle; thus the identity of a germ cell may be very hard or impossible to know if it is taken out of its context, that is, outside the cross-section where it is found. Given the fact that the stages are constantly progressing, stage recognition can also sometimes be complicated by the occurrence of tubule cross-sections in transition between 2 stages (9). Moreover, most staining techniques or sample quality do not allow visualization of subtle differences in cellular structures unique to a particular spermatogenic cell type; therefore, identification is based on association. More precisely, the presence of certain meiotic (spermatocytes) and premeiotic (spermatogonia) germ cell types is always associated with particular postmeiotic (spermatids) germ cell types, as illustrated in Fig. 1, thus the identity of any specific spermatogenic cell can be inferred by association.

Manual stage identification for an entire testicular cross-section that typically contains 100 to 200 seminiferous tubule cross-sections and more than 40 000 cells is laborious and time-consuming. Stage recognition also requires special expertise and a long training. Moreover, the lack of postmeiotic cells in a mutant or a toxicant-exposed mouse makes stage recognition complex and ambiguous for the human eye. For these reasons, we aimed at developing an automated tool for the analysis of mouse testicular histology with the prospect of facilitating reproductive science and the diagnostics of male infertility. Previously, pioneering work in the field developed a computer program to assist male germ cell tracking (10), and methods for automatic segmentation of tubules (11) and cells (12). Subsequently, an automated method for staging using a GATA4 immunostaining was proposed (13). More recent work has employed deep learning that has shown superior performance to other methods in several computer vision tasks (14–16). Similar to our work is an approach that modified ResNet models to segment seminiferous tubules on hematoxylin-eosin (H&E)-stained cross-sections followed by segmentation and classification of 3 cell types in stage VI to III tubules: RS, spermatocytes, and spermatogonia (17). Subsequently the same group published a computerized spermatogenesis staging system that was developed for H&E-stained adult mouse seminiferous tubule cross-sections (18). Computerized spermatogenesis staging consists of 3 modules for automated segmentation of seminiferous tubules followed by classification into 3 stage groups I to V, VI to VIII, and IX to XII, and later subclassification of stage group VI to VIII into VI, VII and VIII, and late VIII.

Here we describe the development and performance of “STAGETOOL.” Besides automated stage recognition (stages I-V, VI-VIII, IX, X-XI, and XII), STAGETOOL can also identify and quantify 8 different categories of spermatogenic cells plus Sertoli cells. We further showcase the use of the tool for automated protein expression analysis. It offers a possibility for large-scale projects in the study of male germ cell differentiation, such as gene expression screening and testicular phenotyping of genetically modified or toxin-exposed mouse models. It operates on 1024 × 1024 pixel images of ×400 magnification containing optimal information for tissue and cellular content analysis, a cross-section of a single seminiferous tubule, and the cells therein. STAGETOOL can also be employed to analyze whole-testis cross-sections. For that, merged whole-testis scans are cut into overlaid smaller images that are iteratively passed to the STAGETOOL. STAGETOOL image predictions are then combined to determine each seminiferous tubule's segmentation, stage classification, and cell-type composition. STAGETOOL also provides other helpful information, for example, cell-type ratios in seminiferous tubule cross-sections for each stage of the seminiferous epithelial cycle.

Compared to previous studies, the work at hand has a few remarkable advantages that are primarily based on the use of DAPI (4′,6-diamidine-2′-phenylindole dihydrochloride) chromatin staining combined with immunofluorescence microscopy for cell and seminiferous epithelial stage recognition and identification. Compared to H&E staining, fluorescent DAPI staining has very little background and even minute differences in chromatin density (euchromatin vs heterochromatin) can be visualized. This allows highly accurate cell identification to be performed only on the basis of nuclear morphology. Moreover, the use of DAPI allows antigen expression profiling where signals from different fluorescent channels can be overlaid with cell and stage predictions. Our DAPI-based approach also enables staging in infertile mouse models where spermatogenesis halts at the round spermatid stage.

## Materials and Methods

### Dataset Description

Wild-type mice of C57BL/6 and mixed backgrounds were maintained under controlled conditions (12 hours dark/12 hours light, temperature, humidity) in individually ventilated cages (Tecniplast) at the Central Animal Laboratory of University of Turku, Turku, Finland. The mice had free access to food and water. Animal husbandry and use were carried out according to Finnish law and following the guidelines of the Ethics of Animal Experimentation in University of Turku in accordance with the Guide for Care and Use of Laboratory Animals. The use of experimental animals in this study was approved by the University of Turku Ethics Committee for animal experiments.

Adult (aged > 8 wk) mouse testes were dissected and fixed in 4% paraformaldehyde overnight at room temperature and embedded in paraffin. Sections (5 µm thick) were cut, deparaffinized (xylene 3 × 3 min, 100% EtOH 2 × 3 min, 96% EtOH 2 × 3 min, 70% EtOH 2 × 3 min, and Milli-Q water 1 × 5 min) and 1) stained with DAPI (1:10 000, Sigma, D9542; 10 min at room temperature) and mounted with ProLong Diamond Antifade Mountant (Thermo Fisher), or 2) processed for immunofluorescent labeling following a previously described protocol (19) with the following primary antibodies: alpha-SMA (1:300; Abcam Inc, ab184675, RRID: AB_2832195; labels peritubular myoid cells), SCP3 (1:200; Abcam Inc, ab150292, RRID: AB_2895074; labels Spc), SOX9 (1:400; Merck-Millipore, AB5535, RRID: AB_2239761; labels Sertoli), SALL4 (1:2000; Abcam Inc, ab29112, RRID: AB_777810; labels Spg), PNA (1:200; Vector Laboratories, RL-1072, RRID: AB_2336642; labels RS + elSpt), USF1 (1:100, SCBT, sc-8983, RRID: AB_2213986; labels Sertoli + Spg), CREM (1:100; SCBT, sc-440, RRID: AB_673599; labels RS), AR (1:200; Abcam Inc, ab133273, RRID: AB_11156085; labels Sertoli); and anti-rabbit secondary antibody A21206 (RRID: AB_2535792; Thermo Fisher Inc). Either citrate buffer (10 mM sodium citrate, pH 6.0) or TE buffer (10 mM Tris-EDTA, pH 9.0) were used for antigen retrieval.

Testis sections from *Spef2* knockout (KO) mice (20) were processed and stained with DAPI as described earlier. Original scans were produced from fluorescently labeled mouse whole-testis cross-sections using a Pannoramic MIDI scanner (3DHistech). A 40× objective lens with 10× ocular was used (×400). The files were converted to TIFF using dedicated software (https://www.3dhistech.com/caseviewer). Image size of mouse testis cross-sections was approximately 4 × 4 mm, which corresponds to 24832 × 24832 pixels (1 px = 0,161 μm). To test the robustness of our processing pipeline, we also used additional images of varying quality originating from collaborators (different sample processing, laboratory, and imaging device).

### Generation of Ground-Truth Data for Seminiferous Tubules

**Figure 2.**
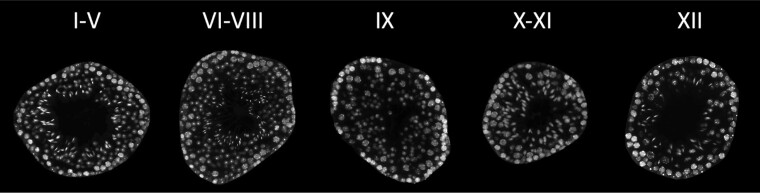
Examples of seminiferous tubule cross-sections representing stages I to V, VI to VIII, IX, X to XI, and XII. Given the synchronous development of spermatogenic cell cohorts, particular types of spermatids are always found together with particular types of spermatogonia and spermatids. This image provides an example cross-section from each stage category.

Four whole-testis cross-sections, plus areas from additional 3 testis cross-sections with rare developmental stages, were selected to generate training and validation data for convolutional neural network models. All these cross-sections originated from different biological replicates. To assist the manual annotation of tubule borders, whole-testis cross-sections were labeled with Alexa Fluor 488-conjugated anti-α smooth-muscle actin (αSMA) antibody. In the adult mouse testis, αSMA is predominantly expressed by peritubular myoid cells, which encase seminiferous tubules (21). Thus, αSMA staining reveals the actual biological tubule borders, which makes the segmentation data very accurate and significantly speeds up the work of manual annotators. Final segmentation data were generated by converting carefully revised tubule border images into a list of tubule contour coordinates, which could be overlaid with the original DAPI-stained images. The tubules that were previously segmented were manually annotated using a VGG Image Annotator tool (https://www.robots.ox.ac.uk/~vgg/software/via/). Specifically, the developmental stages of seminiferous tubules (I-XII; Fig. 1) were divided into 5 categories: I to V, VI to VIII, IX, X to XI, and XII (Fig. 2). The criteria for identification of the stages of the seminiferous epithelial cycle are described in Table 1 and in (2).

**Table 1.** Criteria for identification of epithelial stages

Epithelial stages	Description
I-V	Presence of Spg, pachytene Spc, RS, and late condensed elSpt, which are in bundles. Pachytene Spc are relatively small (I-IV) but gradually grow in size toward stage V.
VI-VIII	elSpt are no longer in bundles and start to align (VI) or are aligned (VII-VIII) at the luminal edge of the epithelium, type B Spg or preleptotene Spc on the basement membrane, pachytene Spc large in size.
IX	Start of spermatid elongation, lack of RS, and late condensed elSpt. Late Spc large and early Spc small.
X-XI	Spermatids are elongated but not fully condensed and have a hooked tip, lack of RS. Late Spc very large and early Spc small in size.
XII	Presence of cells undergoing meiotic divisions (metaphase plates) and/or secondary spermatocytes.

After annotations, DAPI-stained whole-testis cross-sections were sliced into images of 1024 × 1024 pixels. Additionally, the same cross-sections were shifted by 512 × 512 pixels and sliced again to avoid border effects. After slicing, tubule annotation masks that were smaller than 12 000 pixels (∼ 1% of the image area) were removed from the training data. Next, the resulting 3097 training images were augmented by applying random flips and rotations. To further enhance model robustness, blurring and brightness adjustment were introduced to a randomly chosen 20% of the images. As a result, an annotated data set of 27 873 images was created for training and testing the seminiferous tubule segmentation model.

### Generation of Ground-Truth Data for Cell Types

To assist the manual annotation of cell types, DAPI-stained cross-sections were labeled with anti-SALL4 (Spalt-like transcription factor 4) antibody and lectin PNA (peanut agglutinin). SALL4 labels all types of spermatogonia (type A, type intermediate and type B) (22), whereas PNA produces specific labeling patterns for acrosomes in RS and elongating spermatids (2).

Before annotations, 1 DAPI-stained whole-testis cross-section was sliced into 349 images of 1024 × 1024 pixels. An additional 75 images, which contained rare stage XII tubules, were added from 3 other whole-testis cross-sections to balance the number of samples of all cell types in the data set.

The annotations contained 10 cell classes. However, as differentiating between early and late pachytene spermatocytes does not provide meaningful information, these classes were merged. This resulted in 9 cell types, which were selected for classification: Sertoli cells (Sert), spermatogonia (Spg), prepachytene spermatocytes (Spc-prePach; including preleptotene, leptotene, and zygotene spermatocytes), pachytene and diplotene spermatocytes (Spc-Pach), meiotic divisions (m2m), secondary spermatocytes (Spc-sec), RS, early elongating spermatids (elSpt-early), and late elongating spermatids (elSpt-late) (see Fig. 1). Cell types were annotated using the Supervisely online tool (https://supervise.ly). In total, 52 807 cell labels were annotated on 424 images. Finally, the corresponding images with only DAPI staining were fully augmented (flips, brightness, and blur) into 5088 images and 633 684 cell labels.

### Models

We adopted FAIR's (Facebook AI Research) Detectron 2 object detection and segmentation platform version 0.2.1 independently for a model of instance segmentation of seminiferous tubules and a second model for cell-type classification of mouse testis. The resulting 2 models were then combined into a tool that performed consecutive cell and tissue-level analyses on input images (a DAPI-stained mouse testis cross-section).

### Cell Model for Cell-type Classification

We adopted the Faster-RCNN model with ResNeXt-101 as the model's backbone (faster_rcnn_X_101_32 × 8d_FPN_3x.yaml model file), with random initialized weights. The best parameters for the model were empirically determined and set to the following:

cfg.MODEL.ROI_HEADS.BATCH_SIZE_PER_IMAGE = 128
cfg.MODEL.ROI_HEADS.NUM_CLASSES = 10
cfg.MODEL.ROI_HEADS.SCORE_THRESH_TEST = 0.5
cfg.DATALOADER.NUM_WORKERS = 2
cfg.SOLVER.IMS_PER_BATCH = 2
cfg.SOLVER.BASE_LR = 0.0005 (base learning rate)
cfg.SOLVER.WARMUP_ITERS = 500
cfg.SOLVER.MAX_ITER = 30 000
cfg.TEST.DETECTIONS_PER_IMAGE = 10 000

To further increase the sensitivity of cell detection, we rotated the input image during prediction by multiples of 90° and merged the 4 results, discarding overlapping cell predictions (intersection over union [IOU] > 0.5). Also during prediction, 2 of the classes (early and late pachytene spermatocytes), were fused into 1 (pachytene and diplotene spermatocytes, Spc-Pach), as explained earlier.

### Tubule Model for Seminiferous Tubule Segmentation and Classification

For sample segmentation of seminiferous tubule cross-section images, we employed Mask-RCNN, with ResNet-50() as the model's backbone (mask_rcnn_R_50_FPN_3x.yaml model file), pretrained on ImageNet. The best model parameters were empirically determined and set to the following:

cfg.MODEL.ANCHOR_GENERATOR.SIZES = [[128, 256, 512, 1024]] (base box size)
cfg.SOLVER.BASE_LR = 0.0005 (base learning rate)
cfg.MODEL.ROI_HEADS.SCORE_THRESH_TEST = 0.3 (Threshold on inference)
cfg.MODEL.ROI_HEADS.NUM_CLASSES = 5

### STAGETOOL Model for Simultaneous Cell and Tissue-level Analysis

**Figure 3.**
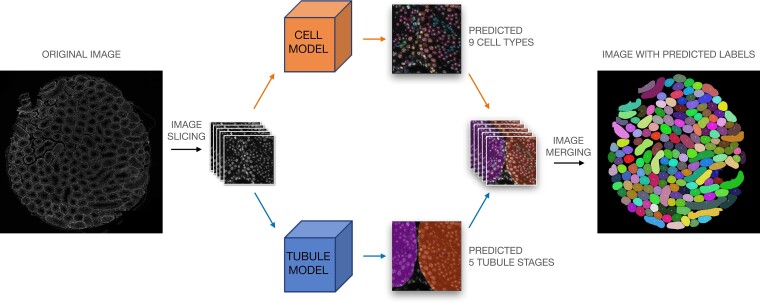
STAGETOOL scheme: A whole-testis DAPI-stained cross-section image was sliced into a collection of 1024 × 1024 images. Then each image in the collection went through 2 different neural network models: cell and tubule model. The cell model detected and classified the seminiferous epithelial cells into 9 different cell types. The tubule model detected, segmented, and classified tubules into 5 developmental stage categories. Finally, the whole-testis cross-section was composed again including all the tubule and cell information.

STAGETOOL is a combination of the cell and tubule models described earlier. Its broad pipeline is illustrated in Fig. 3. A whole-testis cross-section image is cropped to 1024 × 1024 pixels, 400× magnified mouse testis images, which were an appropriate input for the neural networks. To avoid losing information at the borders, the cross-section image was shifted by 512 × 512 pixels and sliced identically again, duplicating the number of input images. Every input image was passed to both cell and tubule models, and the outputs were merged into a combined report.

For cell-type predictions, each image was analyzed at every 90° rotation angle altogether 4 times, and the results were merged by weighted majority vote. When stitching together the mosaic of half-overlapping images, predictions for the same cells (IOU ≥ 0.4) were merged again by weighted majority vote. Second-best predictions for merged cells were also collected. Finally, the predictions of the cell and tubule models were combined. Because stage XII is often a mixture of stages XII and I, 2 merging rules were applied to the tubule and cell model predictions. First, if the cell model did not detect any metaphase plates or secondary spermatocytes in the tubule predicted to be stage XII by the tubule model, that tubule was instead stage I to V. Secondly, if metaphase plates or secondary spermatocytes were found in a tubule that was not predicted to be stage XII, their labels were replaced by the second-best predictions (if there were any) or deleted.

**Figure 4.**
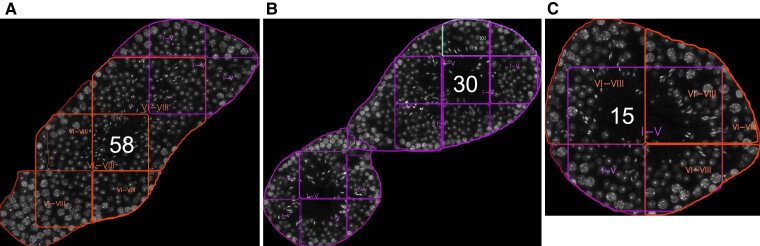
Visualization of subpredictions for tubule cross-sections. Cross-sections of tubules A, 58; B, 30; and C, 15 from the whole-testis cross-section analysis. Each tubule is represented by several overlapping images, which have individually predicted tubule borders and classes. A weighted majority algorithm merges the predicted classes for each tubule, taking into account the prediction confidences, predicted mask areas, and a set of stage-specific rules.

For the predictions of the developmental stages of whole tubules, all predicted tubule masks were mapped by many-to-one logic to each tubule (Fig. 4). Sometimes manual curation was required to remove unsuitable tubules, which may often occur in whole-testis cross-section images and cause segmentation errors. On average, a union of 8 predicted masks made 1 tubule. For each mask, the confidence value of the predicted class was multiplied by the mask's percentage-area in the tubule. This increased the prediction weight of larger masks. These confidence values for the predicted classes were then multiplied among the masks to find the best label for the tubule. The final report contains an overlay of the input image with all detected objects (see Fig. 3). Additionally, detailed information about the segmented seminiferous tubules can be provided in a tabular format (Table 2), including the developmental stages and their respective cell-type composition.

**Table 2.** STAGETOOL output summary: cell labels vs epithelial stages

Cell class	I-V	VI-VIII	IX	X-XI	XII
Sert	1024	291	311	150	157
Spg	711	121	86	51	77
Spc-prePach	219	631	1235	521	458
Spc-Pach	3565	1038	1277	640	188
Spc-sec	16	0	0	0	436
m2m	29	0	0	0	177
RS	9777	2692	104	0	223
elSpt-early	0	5	3153	1385	17
elSpt-late	7586	1871	93	398	1757

Abbreviations: m2m, meiotic divisions; RS, round spermatids.

## Results

### Wild-type Mouse DAPI-stained Testis Cross-section Image Analysis

**Figure 5.**
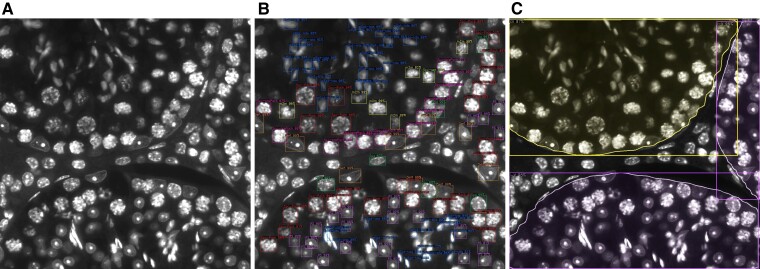
Sample results produced by the 2 models. A, Original image; B, cell model output; and C, tubule model output.

We first evaluated individually the performance of the 2 models (cell and tubule model) that comprise STAGETOOL on test images from the DAPI-stained annotated data set. Subsequently we showcase STAGETOOL stage classification on a whole-testis cross-section. Examples of STAGETOOL outputs are provided in Fig. 5.

### Cell Model Evaluation

The cell model was evaluated on 71 left out images from the DAPI-stained annotated data set. Some examples of the qualitative results are shown in Fig. 5B. Regarding quantitative results, first a metric typically employed on object detection tasks was calculated: mean average precision (AP), as defined in the COCO object detection challenge (23). The model obtained an AP50 of 78.99 and AP75 of 50.00 (24, 25). Mean AP reflects how much the set of predicted boxes corresponds to the set of ground-truth boxes on average over all the classes. However, beyond exact box coordinates and size, it is essential to evaluate how well the model can detect and classify cells for each category. Therefore, the IOU (Jaccard Index) threshold was fixed to 0.5 to match predictions and ground truth to assess classification performance. Using these conditions, positive predictive value, sensitivity, and F1 score were measured for each cell class. Table 3 provides the summary statistics for each cell category. When averaged across classes, the positive predictive value is 0.9607, pointing out that the model could differentiate appropriately between classes. Sensitivity was in general high with an average across class of 0.8402, but lower than the positive predictive value. elSpt-late stood out by their lower sensitivity with around 69% of the ground labels detected. For elSpt-late the lower percentage of detection might have been due to their shape, ill suited to fit a box, their small size, and the fact that they are often bundled together, causing several prediction boxes to overlap. Thus, the neural network might have considered overlapping predictions from the same cell and eliminated some of them with the nonmaximum suppression algorithm.

**Table 3.** Cell model quantitative results at 0.5 intersection over union

Cell class	Positive predictive value	Sensitivity	F1-score
Sert	0.9934	0.9700	0.9815
Spg	0.9152	0.7593	0.8300
Spc-prePach	0.9390	0.8964	0.9173
Spc-Pach	0.9782	0.9352	0.9562
Spc-sec	0.9906	0.8203	0.8974
m2m	0.9500	0.8261	0.8837
RS	0.9947	0.9033	0.9468
elSpt-early	0.9243	0.7680	0.8389
elSpt-late	0.9611	0.6829	0.7985

Abbreviations: m2m, meiotic divisions; RS, round spermatids.

**Figure 6.**
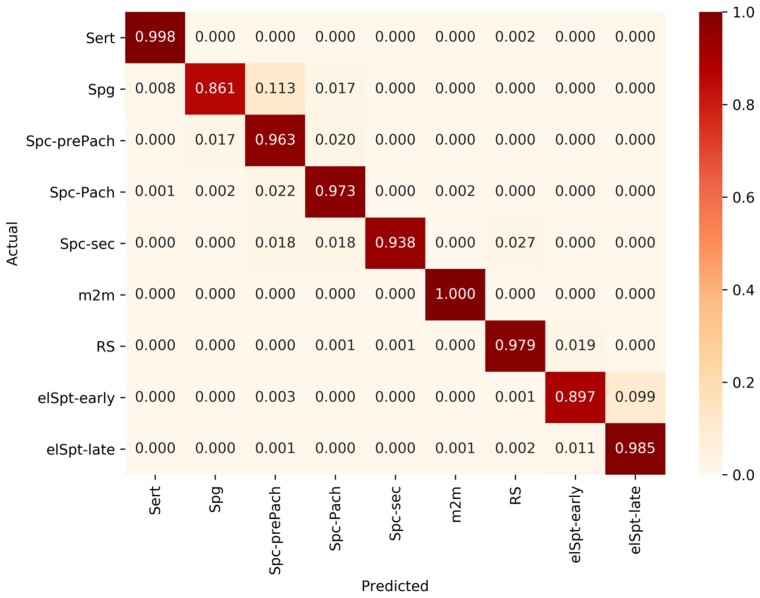
Confusion matrix for the 9 seminiferous epithelial cell types.

A confusion matrix (Fig. 6) shows the incidence of erroneous cell labels for any 9 particular seminiferous epithelial cell types. Understandably, the most common mistakes were between 1) Spc-prePach and Spg, which are occasionally very hard to discern without an antibody staining; and 2) elSpt-early and elSpt-late, where the former gradually convert to the latter.

### Tubule Model Evaluation

For tubule model evaluation we used 242 left out images from the annotated data set. Some examples of the qualitative results are shown in Fig. 5C. The quantitative results for 4-pixel level metrics (24) for the tubule model are shown in Table 4. Although given the image size, the model typically receives only partial information about any given tubule, the model obtains a pixel accuracy of 0.918. The mean IOU metric of 0.74 was caused mainly by stage X to XI tubules predicted to be stage IX, which caused an IOU below 0.5 for the class. In the next section the model is evaluated at the whole-testis level to obtain a better assessment of its performance.

**Table 4.** Quantitative results for tubule instance segmentation

Pixel accuracy	0.9180
Mean accuracy	0.8551
Mean IU	0.7423
Frequency weight IU	0.8569

Abbreviation: IU, intersection over union.

### Whole-testis Cross-section Evaluation

**Figure 7.**
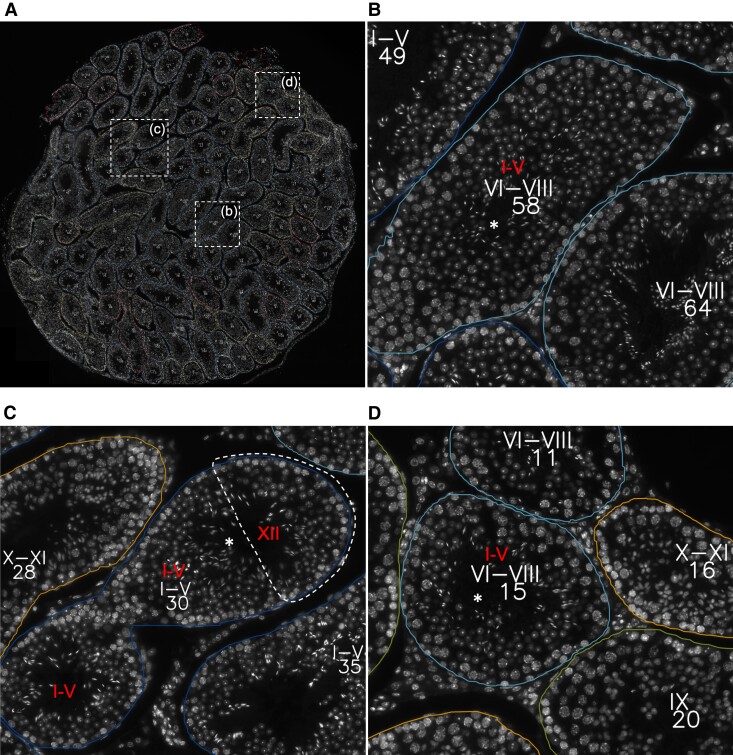
Whole-testis tubular level analysis. A, A whole-testis cross-section with STAGETOOL-derived staging data; white dotted rectangles are shown as higher magnification insets in B to D. B, An example of a borderline stage V to VI tubule (asterisk, tubule 58). C, Tubule 30 (asterisk) is a longitudinal cross-section of a tubule in which the left and middle part are in stage I to V, whereas the right corner represents stage XII. D, Tubule 15 (asterisk) shows the only actual conflict between human annotation and computer prediction. Human annotation, if in conflict with the computer prediction, is shown in red. A high-resolution image of the same whole-testis cross-section analysis with cellular and tubular labels is provided online (https://dx.doi.org/10.21227/em1y-9j15). Subpredictions for individual images comprising tubules 58, 30, and 15 are shown in Fig. 4.

To perform the analysis at the whole-testis level, a testis cross-section was divided into 1024 × 1024 overlapping images for model input. The output predictions were merged and the whole-testis cross-section image was recompiled, with the annotated tubular and cellular objects. Fig. 7 illustrates a representative example of such an analysis at the tubular level, while a high-resolution image of whole-testis cross-section analysis with cellular and tubular labels is provided online (https://dx.doi.org/10.21227/em1y-9j15). STAGETOOL output was compared to an assessment of an experienced human histologist (15 years of experience). The testis cross-section (Fig. 7A) consisted of 112 tubular cross-sections and in 107 cases (96%; Table 5) there was equivalence between the human and computer predictions, and discrepancy in 5 cases (4%). In particular, in 4 out of 5 five cases the discrepancy was between stages I to V vs VI to VIII and in 1 case XII vs I to V. Further in-depth analysis revealed that in 3 of the former cases (tubule 58 as an example; Figs. 4A and 7B) the disagreements concerned stages V and VI where either stage category I to V or VI to VIII were correct. In another case (Fig. 7C; tubule 30) the computer prediction (I-V) was in conflict with the human annotation (XII). However, this was a longitudinal tubule cross-section that mostly presents stage I but with the right corner (dotted line in Fig. 7C) being in stage XII, as also revealed by image-level output (Fig. 4B). Thus, only in one case (Fig. 7D; tubule 15) was the computer prediction biologically wrong, although image-level analysis (Fig. 4C) also revealed uncertainty in the model staging but the voting algorithm selected stage VI to VIII over I to V. Altogether, these data show that the accuracy of STAGETOOL in whole-testis tubular analysis is 99.1%.

**Table 5.** Comparison of human annotations vs computer predictions (in parentheses when borderline cases are taken into account) for the whole-testis cross-section provided in Fig. 7A

Total No. of tubules	Agreement human vs computer	Disagreement human vs computer
112	107 (111) 95.6% (99.1%)	5 (1) 4.5% (0.9%)

**Figure 8.**
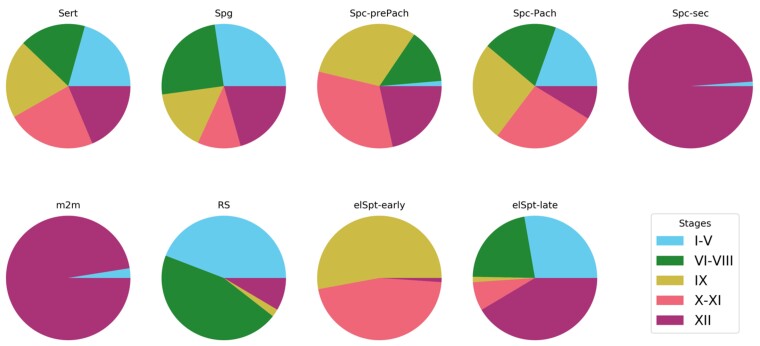
Distribution of cell labels in different epithelial stages as extracted from STAGETOOL output. As expected, Sertoli cells were evenly distributed across the stages, whereas secondary spermatocytes (Spc-sec) and metaphase plates (m2m) were seen in stage XII, and occasionally in stage I to V (see Fig. 7C for explanation). Distribution of cell types in stage categories were accurate and biologically correct.

To provide a numerical analysis of STAGETOOL performance, we studied the distribution of cell labels at different seminiferous epithelial stages (Fig. 8). STAGETOOL faithfully recognizes the 9 seminiferous epithelial cell types in 5 epithelial stage categories. The distributions are biologically correct and expected (see Fig. 1).

### STAGETOOL Robustness and Knockout Testis Analysis

After measuring STAGETOOL performance in our annotated data set, we proceeded to test the robustness of STAGETOOL and determine its detection limits. To this end, the model was retrained with all the annotated data available, including the leftover test set images. Subsequently its performance was tested on several seminiferous tubule cross-section images from KO mice with a spermatogenic defect. The images originated from different laboratories and were generated by different instruments and at different mouse ages. Image quality was disparate, and the resolution differed from the training data in a few cases. All images were converted to grayscale since it is required by STAGETOOL.

**Figure 9.**
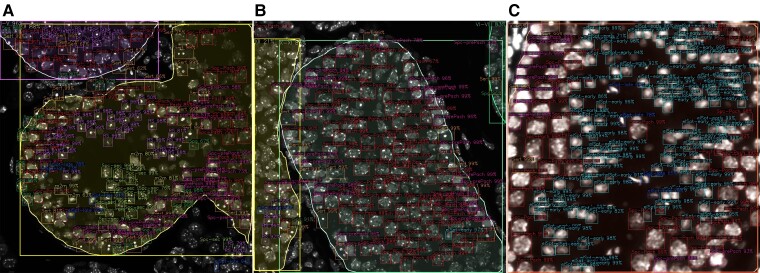
STAGETOOL output images for A and B, *Miwi* knockout (KO) and C, *Spef2* KO mouse seminiferous tubule cross-sections. Two representative examples of STAGETOOL output for *Miwi* KO seminiferous tubules at stages A, I to V and XII, and B, X and XI. Despite the lack of elongating spermatids, STAGETOOL correctly recognized the stages and cell types therein. C, A representative example of STAGETOOL output for low-resolution and over-exposed *Spef2* KO seminiferous tubules at stage IX.

STAGETOOL was applied to several good-quality seminiferous tubule cross-section confocal images from *Miwi* KO mice, which are characterized by spermatogenic arrest at the RS stage and a lack of elongating spermatids (26). Lack of elongating spermatids makes staging for *Miwi* KO testis challenging because it is primarily the presence and intraepithelial distribution of elongating spermatids that define the stages for a human histologist. Fig. 9A and 9B illustrate representative examples of STAGETOOL output. Although meaningful general statistics could not be extracted because of a low number of images, of the 856 cells predicted on *Miwi* KO mice, only 7 were incorrectly categorized as elongating spermatids (which do not exist in these mice). Overall, after visual inspection, cell categorization was in line with the results obtained in the annotated data set. More notably, tubule staging was correct in the *Miwi* KO mouse line (see Fig. 9A and 9B), indicating that the model does not depend on the presence of elongating spermatids to determine seminiferous epithelial stages, like a human histologist typically does.

We also tested several images of paraffin-embedded seminiferous tubule cross-sections from *Spef2* KO mice (Fig. 9C). All these images were of low resolution and partially overexposed. *Spef2* KO mice are characterized by elongating spermatids of abnormal head and short tail defects (20). STAGETOOL was nonetheless able to characterize cell types and tubule stages highly precisely.

### STAGETOOL for Antibody Expression Profiling

Having evaluated the performance and robustness of STAGETOOL, we wanted to evaluate whether STAGETOOL could be used for automated profiling of antigen expression by combining DAPI staining with fluorescent antibody labeling. Many antigens have restricted expression in spermatogenic cells, and their expression is often limited to particular cell types and specific stages of the seminiferous epithelial cycle. Applying STAGETOOL for the assessment of cell type or stage-specific antigen expression would be a remarkable milestone because it would enable reliable analysis of antigen expression in the seminiferous tubules without prior experience in testicular histology or staging.

**Figure 10.**
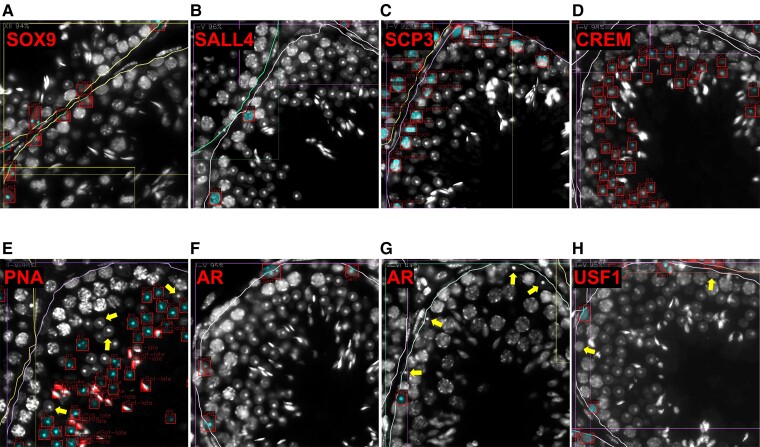
Snapshots of STAGETOOL output for whole-testis automated antigen expression analysis. Signals from antibody stainings (red) are overlaid with 4′,6-diamidine-2′-phenylindole dihydrochloride (DAPI) staining (white) and STAGETOOL-derived cell type and stage predictions to acquire cell type and stage-specific expression pattern for different antigens. The DAPI signal from respective antigen-positive cells is highlighted with cyan. A, SOX9 (red) is expressed uniformly in Sertoli cells. B, SALL4 (red) is expressed in all spermatogonia. C, SCP3 (red) staining is seen in prepachytene and pachytene spermatocytes and a subset of spermatogonia. D, CREM localizes to round spermatids (RS). E, PNA (red) stains the acrosomes of round and elongating spermatids. In this stage (II-IV), the acrosomic vesicles of RS are small and therefore there are many RS that stain negatively for PNA (some indicated with yellow arrows). F and G, Androgen receptor (AR; red) is expressed in Sertoli cells in a stage-dependent manner. In stages I to V, F, AR signal is observed in all Sertoli cells, whereas in stages X to XI, G, AR signal is high enough to qualify as positive only in a subset of Sertoli cells, resulting in stage-specificity for AR expression as shown in Fig.11. Yellow arrows indicate Sertoli cells with subthreshold level of AR expression. H, USF1 (red) is expressed in Sertoli cells and a subset of spermatogonia. Yellow arrows indicate USF1-negative intermediate/type B spermatogonia.

To evaluate the feasibility of such an analysis, we stained mouse testis cross-sections with DAPI and the following antibodies: SOX9 (SRY-box transcription factor 9), AR (androgen receptor), USF1 (upstream stimulatory factor 1), SALL4, SCP3 (synaptonemal complex protein 3), CREM (cAMP responsive element modulator), and PNA (Fig. 10). SOX9, AR, and USF1 are expressed in Sertoli cells, but while SOX9 (27) and USF1 (28) are expressed at an equally high level in all Sertoli cells independent of the epithelial stage, AR expression is seen at a high level in Sertoli cells at early-to-mid stages and lower in late stages (IX-XII) (29, 30). USF1 is also expressed in undifferentiated and early differentiating spermatogonia (28), whereas SALL4 is a pan-spermatogonial marker (22). SCP3 is needed for synaptonemal complex assembly, and therefore expressed in spermatocytes (31, 32). CREM is a transcription factor specifically expressed in RS (33–35), whereas PNA stains the acrosomes of RS and elongating spermatids (2).

**Figure 11.**
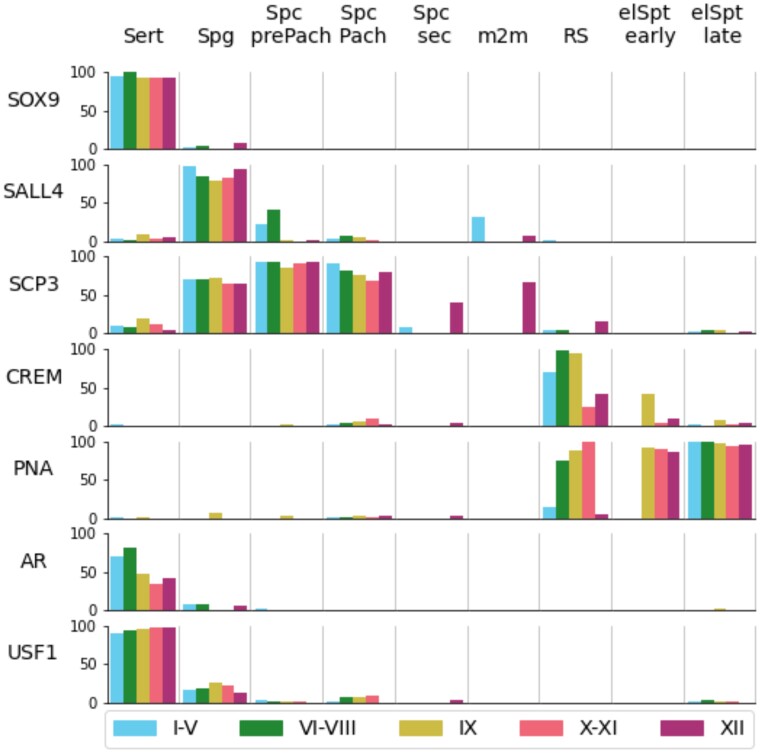
STAGETOOL-derived antigen expression profiles for several proteins expressed in Sertoli cells and spermatogenic cells. Percentage (bar height) of predicted cells type (columns) of a given tubular stage (color) labeled by the corresponding antibody (row). SOX9 and SALL4 were uniformly expressed by Sertoli cells and spermatogonia, respectively. SCP3 staining was observed in all primary spermatocytes and a subset of spermatogonia, whereas CREM was seen only in round spermatids (RS). PNA localized to postmeiotic cells that have the acrosome, note well that acrosomes in early RS are absent (stage I) or very small in size (II-IV) (2). Androgen receptor (AR) was most highly expressed in early-to-mid stages, whereas upstream stimulatory factor 1 (USF1) was expressed stage-independently in Sertoli plus a subset of spermatogonia. STAGETOOL-derived expression profiles were as expected and biologically accurate. Background staining for some of the antigens (eg, Sertoli and early spermatocyte staining for SALL4) was primarily due to use of ovals to define cell boundaries. Occasionally areas from more than just one cell ended up in ovals causing a leakage of spermatogonial signal to neighboring early spermatocyte, for instance. Unspecific staining only slightly biased the analysis.

To obtain the antigen expression profiles, STAGETOOL was first applied to the cross-section image containing only DAPI information. Subsequently, the predicted tubules and cells were overlaid with the immunofluorescent image from the antibody stainings and the luminosity threshold was fixed empirically for each fluorescent marker (see “Materials and Methods”). The predicted cells with mean luminosity above the threshold and 65% of the pixels above half threshold were considered positive for antibody expression. STAGETOOL-derived antigen expression data confirmed the anticipated cell type-specific expression pattern for SOX9 (in Sertoli cells; Fig. 10A), SALL4 (Spg; Fig. 10B), SCP3 (Spc; Fig. 10C), CREM (RS; Fig. 10D), and PNA (RS and elongating spermatids; Fig. 10E). STAGETOOL was also able to record stage-specific expression for AR (Fig. 10F and 10G) in Sertoli cells of early-to-mid epithelial cycle and USF1 expression (Fig. 10H) in only a subset of spermatogonia but uniformly in all Sertoli cells independent of the stage of seminiferous epithelial cycle. These predictions were biologically accurate and in agreement with the literature. Fig. 11 summarizes the STAGETOOL-derived expression profile for the studied antigens.

## Discussion

The aim of this study was to develop a machine learning–based tool for the automated staging of fluorescent-labeled seminiferous tubules in mouse testis cross-sections and identification of seminiferous epithelial cell types. STAGETOOL provides adequate, quick, and scalable analysis of the microscopy images of DAPI-stained mouse testis cross-sections both at the cellular and tubular levels. To our knowledge, the present study is the first attempt for automated stage recognition of mammalian seminiferous epithelial cycle based on use of a fluorescent dye, DAPI, to stain the chromatin in cells within the testicular cross-sections. The cell and epithelial stage recognition relies heavily on nuclear size/morphology and chromatin organization in different cell types, emphasizing the importance of using a chromatin stain. Furthermore, the data do not suffer from background signal from cell cytoplasm and extracellular matrix, which further facilitates the analysis. The use of fluorescent chromatin dye also enables simultaneous co-immunofluorescent staining of several proteins to characterize their expression patterns by combining the immunofluorescence imaging with cell and stage-level data.

To assess spermatogenesis in detail, the analysis of both stage organization of the seminiferous epithelium and its cellular composition are required. A clear benefit of STAGETOOL is that it combines these 2 analyses. The accuracy for whole-testis tubular stage analysis is 99%, so it provides a very reliable tool for the recognition of the 5 stage pools (I-V, VI-VIII, X, X-XI, and XII). In the future, we aim to expand the model to recognize all 12 stages (I-XII) of the mouse seminiferous epithelial cycle, even though staging into these 5 categories is typically sufficient in a laboratory conducting male reproductive biology research. Our cell model obtains an average accuracy of 0.96 for 9 different seminiferous epithelial cell types. This high performance was achieved through tedious manual annotation of nearly 53 000 cells. We believe that the performance of STAGETOOL can be pushed even further after retraining with the whole corpus of training, testing, and validation data, which will be performed during the continuous updating of the final version.

In addition to the wild-type spermatogenesis, we also showcase the functionality of STAGETOOL for analysis of genetically modified mouse models with a spermatogenic defect. Stage recognition in such mice may be challenging for human histologists because of the lack or reduced numbers of specific spermatogenic cell types. However, STAGETOOL was able to perform stage and cell-type recognition in *Miwi* and *Spef2* KO mouse models very accurately despite the complete lack of elongating spermatids (*Miwi* KO) or disrupted spermatid elongation (*Spef2* KO). We are not aware of the histomorphological features that the model uses to achieve this.

While STAGETOOL is fully automated, the whole-testis cross-section image analysis tool requires some manual curation to remove unsuitable seminiferous tubules from the segmentation output image. This is because the tubular arrangement of the whole testis often contains artifacts from sample preparation or longitudinally cut tubules, which may interfere with downstream analyses. Therefore, we can call the whole-testis approach only semiautomated, whereas on select 1024 × 1024 images STAGETOOL operates fully automatedly. Nonetheless, semiautomated whole-testis cross-section analysis offers a possibility for large-scale projects in male germ cell differentiation, such as gene expression screening and testicular phenotyping of genetically modified mouse models. When tested on Google Colab free GPU, it takes 1.5-5 seconds to fully analyze a 1024 × 1024 pixel image. A whole-testis cross-section, with approximately 600 images, takes then 15 to 50 minutes, accordingly. Manual curation of whole-testis cross-section analyses depends on the image quality and takes an additional 5 to 15 minutes. Antibody expression profiling for whole-testis cross-sections (selecting signal thresholds for the antibody-labeled image) takes 10 to 30 minutes.

In humans and some other primates, the stages are organized differently from rodents, and each seminiferous tubule cross-section can host more than one stage (36). Therefore, STAGETOOL cannot be directly applied to the analysis of human testis. However, the alarming adverse trends in male reproductive health and fertility (37) emphasize the importance of extending STAGETOOL to tackle human spermatogenesis. Although 10% to 15% of couples suffer from infertility (World Health Organization, 2017), the etiology of male factor infertility, contributing to half the cause, is unknown in 70% of cases (38). Therefore, there is a compelling need for better diagnostic tools for the study male infertility and subfertility. This study will pave the way for developing an automated analysis tool for characterizing spermatogenic failure in humans, which would facilitate counseling and selection of treatment strategies for male infertility and shed light on the underlying causes.

## Data Availability

Some or all data sets generated during and/or analyzed during the present study are not publicly available but are available from the corresponding author on reasonable request.

## Abbreviations

αSMA: α smooth-muscle actin
AP: average precision
DAPI: 4′,6-diamidine-2′-phenylindole dihydrochloride
elSpt-early: early elongating spermatids
elSpt-late: late elongating spermatids
H&E: hematoxylin-eosin
KO: knockout
IOU: intersection over union
m2m: meiotic divisions
PNA: peanut agglutinin
RS: round spermatids
SALL4: Spalt-like transcription factor 4
Sert: Sertoli cells
Spc-Pach: pachytene and diplotene spermatocytes
Spc-prePach: prepachytene spermatocytes
Spc-sec: secondary spermatocytes
Spg: spermatogonia

## References

[1] Mäkelä JA , Toppari J. Spermatogenesis. In: Simoni M, Huhtaniemi I, eds. Endocrinology of the Testis and Male Reproduction. 1st ed. Springer International Publishing; 2017:1–39.

[2] Mäkelä JA , Cisneros-Montalvo S, Lehtiniemi T, et al Transillumination-assisted dissection of specific stages of the mouse seminiferous epithelial cycle for downstream immunostaining analyses. J Vis Exp. 2020; 2020(164):e61800. doi: 10.3791/61800 33104058

[3] Mäkelä JA , Toppari J. Testis physiology: seminiferous cycle. In: Jégou B, Skinner MK, eds. Encyclopedia of Reproduction. 2nd ed. Academic Press; 2018:134–144.

[4] Muciaccia B , Boitani C, Berloco BP, et al Novel stage classification of human spermatogenesis based on acrosome development. Biol Reprod. 2013;89(3):60. 23946533 10.1095/biolreprod.113.111682

[5] Oakberg EF . Duration of spermatogenesis in the mouse and timing of stages of the cycle of the seminiferous epithelium. Am J Anat. 1956;99(3):507–516. 13402729 10.1002/aja.1000990307

[6] Hess RA , de Franca LR. Spermatogenesis and cycle of the seminiferous epithelium. In: Cheng CY, ed. Molecular Mechanisms in Spermatogenesis. Vol. 636, Advances in Experimental Medicine and Biology. Springer, 2009:1–15. 10.1007/978-0-387-09597-4_1 19856159

[7] Meistrich ML , Hess RA. Assessment of Spermatogenesis Through Staging of Seminiferous Tubules BT—Spermatogenesis: Methods and Protocols. In: Carrell DT, Aston KI, eds. Humana Press; 2013:299–307. 10.1007/978-1-62703-038-0_27 22992924

[8] Virtanen HE , Jørgensen N, Toppari J. Semen quality in the 21st century. Nat Rev. 2017;14(2):120–130. 10.1038/nrurol.2016.261 28050014

[9] Hess RA . Quantitative and qualitative characteristics of the stages and transitions in the cycle of the rat seminiferous epithelium: light microscopic observations of perfusion-fixed and plastic-embedded testes. Biol Reprod. 1990;43(3):525–542. 2271734 10.1095/biolreprod43.3.525

[10] Hess RA , Chen P. Computer tracking of germ cells in the cycle of the seminiferous epithelium and prediction of changes in cycle duration in animals commonly used in reproductive biology and toxicology. J Androl. 1992;13(3):185–190. 1601739

[11] Fakhrzadeh A , Spörndly-Nees E, Holm L, Hendriks CLL. Analyzing tubular tissue in histopathological thin sections. In: International Conference on Digital Image Computing Techniques and Applications (DICTA). 2012:1–6.

[12] Fakhrzadeh A , Spörndly-Nees E, Holm L, Hendriks CLL. Epithelial cell segmentation in histological images of testicular tissue using graph-cut. In: International Conference on Image Analysis and Processing; 2013:201–208.

[13] Fakhrzadeh A , Spörndly-Nees E, Ekstedt E, Holm L, Luengo Hendriks CL. New computerized staging method to analyze mink testicular tissue in environmental research. Environ Toxicol Chem. 2017;36(1):156–164. 27271123 10.1002/etc.3517

[14] Goodfellow I , Bengio Y, Courville A. Deep Learning. MIT Press; 2016.

[15] Lee S , Fu C, Salama P, Dunn KW, Delp EJ. Tubule segmentation of fluorescence microscopy images based on convolutional neural networks with inhomogeneity correction. 2018:1–8.

[16] Kao C . *A Deep Learning Architecture For Histology Image Classification*. PhD thesis. University of North Carolina at Chapel Hill, 2018. 30689332

[17] Xu J , Lu H, Li H, Wang X, Madabhushi A, Xu Y. Histopathological Image Analysis on Mouse Testes for Automated Staging of Mouse Seminiferous Tubule. ECDP; 2019.

[18] Xu J , Lu H, Li H, et al Computerized spermatogenesis staging (CSS) of mouse testis sections via quantitative histomorphological analysis. Med Image Anal. 2021;70:101835. 33676102 10.1016/j.media.2020.101835 PMC8046964

[19] Yadav RP , Mäkelä JA, Hyssälä H, Cisneros-Montalvo S, Kotaja N. DICER regulates the expression of major satellite repeat transcripts and meiotic chromosome segregation during spermatogenesis. Nucleic Acids Res. 2020;48(13):7135–7153. 32484548 10.1093/nar/gkaa460 PMC7367195

[20] Lehti MS , Zhang FP, Kotaja N, Sironen A. SPEF2 functions in microtubule-mediated transport in elongating spermatids to ensure proper male germ cell differentiation. Development. 2017;144(14):2683–2693. 28619825 10.1242/dev.152108

[21] Lokka E , Lintukorpi L, Cisneros-Montalvo S, et al Generation, localization and functions of macrophages during the development of testis. Nat Commun. 2020;11(1):4375. 32873797 10.1038/s41467-020-18206-0 PMC7463013

[22] Chan AL , La HM, Legrand JMD, et al Germline stem cell activity is sustained by SALL4-dependent silencing of distinct tumor suppressor genes. Stem Cell Reports. 2017;9(3):956–971. 28867346 10.1016/j.stemcr.2017.08.001 PMC5599261

[23] Padilla R , Netto SL, da Silva EAB. A survey on performance metrics for object-detection algorithms. In: 2020 International Conference on Systems, Signals and Image Processing (IWSSIP). 2020:237–242.

[24] Long J , Shelhamer E, Darrell T. Fully convolutional networks for semantic segmentation. In: 2015 IEEE Conference on Computer Vision and Pattern Recognition (CVPR). 2015:3431–3440.

[25] Caicedo JC , Goodman A, Karhohs KW, et al Nucleus segmentation across imaging experiments: the 2018 Data Science Bowl. Nat Methods. 2019;16(12):1247–1253. 31636459 10.1038/s41592-019-0612-7 PMC6919559

[26] Deng W , Lin H. miwi, A murine homolog of piwi, encodes a cytoplasmic protein essential for spermatogenesis. Dev Cell. 2002;2(6):819–830. 12062093 10.1016/s1534-5807(02)00165-x

[27] da Silva SM , Hacker A, Harley V, Goodfellow P, Swain A, Lovell-Badge R. Sox9 expression during gonadal development implies a conserved role for the gene in testis differentiation in mammals and birds. Nat Genet. 1996;14(1):62–68. 8782821 10.1038/ng0996-62

[28] Faisal I , Cisneros-Montalvo S, Hamer G, et al Transcription factor USF1 is required for maintenance of germline stem cells in male mice. Endocrinology. 2019;160(5):1119–1136. 30759202 10.1210/en.2018-01088

[29] Domanskyi A , Zhang FP, Nurmio M, Palvimo JJ, Toppari J, Jänne OA. Expression and localization of androgen receptor-interacting protein-4 in the testis. Am J Physiol Endocrinol Metab. 2007;292(2):E513–E522. 17003240 10.1152/ajpendo.00287.2006

[30] Bremner WJ , Millar MR, Sharpe RM, Saunders PT. Immunohistochemical localization of androgen receptors in the rat testis: evidence for stage-dependent expression and regulation by androgens. Endocrinology. 1994;135(3):1227–1234. 8070367 10.1210/endo.135.3.8070367

[31] Yuan L , Liu JG, Zhao J, Brundell E, Daneholt B, Höög C. The murine SCP3 gene is required for synaptonemal complex assembly, chromosome synapsis, and male fertility. Mol Cell. 2000;5(1):73–83. 10678170 10.1016/s1097-2765(00)80404-9

[32] Park M , Lee Y, Jang H, et al SOHLH2 is essential for synaptonemal complex formation during spermatogenesis in early postnatal mouse testes. Sci Rep. 2016;6(1):20980. 26869299 10.1038/srep20980 PMC4751484

[33] Nantel F , Monaco L, Foulkes NS, et al Spermiogenesis deficiency and germ-cell apoptosis in CREM-mutant mice. Nature. 1996;380(6570):159–162. 8600390 10.1038/380159a0

[34] Blendy JA , Kaestner KH, Weinbauer GF, Nieschlag E, Schütz G. Severe impairment of spermatogenesis in mice lacking the CREM gene. Nature. 1996;380(6570):162–165. 8600391 10.1038/380162a0

[35] Behr R , Weinbauer GF. cAMP response element modulator (CREM): an essential factor for spermatogenesis in primates? Int J Androl. 2001;24(3):126–135. 11380701 10.1046/j.1365-2605.2001.00277.x

[36] Wistuba J , Schrod A, Greve B, et al Organization of seminiferous epithelium in primates: relationship to spermatogenic efficiency, phylogeny, and mating system. Biol Reprod. 2003;69(2):582–591. 12700190 10.1095/biolreprod.103.015925

[37] Skakkebaek NE , Rajpert-De Meyts E, Buck Louis GM, et al Male reproductive disorders and fertility trends: influences of environment and genetic susceptibility. Physiol Rev. 2016;96(1):55–97. 26582516 10.1152/physrev.00017.2015 PMC4698396

[38] Tüttelmann F , Ruckert C, Röpke A. Disorders of spermatogenesis: perspectives for novel genetic diagnostics after 20 years of unchanged routine. Med Genet. 2018;30(1):12–20. 29527098 10.1007/s11825-018-0181-7 PMC5838132

